# The unfolding story of dying tumor cells during cancer treatment

**DOI:** 10.3389/fimmu.2023.1073561

**Published:** 2023-03-13

**Authors:** Sijia He, Qian Huang, Jin Cheng

**Affiliations:** Cancer Center, Shanghai General Hospital, Shanghai Jiao Tong University School of Medicine, Shanghai, China

**Keywords:** cancer, cell death, tumor immune microenvironment, cancer treatment, immunotherapy

## Abstract

Generally, the demise of cancer cells in different ways enables the body to clear these harmful cells. However, cancer cells obtain unlimited replication and immortality from successful circumvention of cell death *via* various mechanisms. Some evidence suggests that treatment-induced dying tumor cells even promote cancer progression. Notably, therapeutic interventions to harness the immune system against tumor cells have shown complicated influences in clinics. Herein, there is an urgent need to clarify the underlying mechanisms that influence the outcome and regulation of the immune system during cancer treatment. In this review, we provide an account on the cell death modes and the relationship between dying tumor cells with tumor immune microenvironment during cancer treatment, focusing on immunotherapy, from mechanistic standpoint to emerging limitations and future directions.

## 1 Introduction

Cancer is characterized by uncontrolled cell proliferation and lack of cell death, which has become a major threat to human health. Usually, treatment options for patients with cancers depend on the stage and type of cancer, which include surgical removal, chemotherapy, radiotherapy, targeted therapy and immunotherapy. It is worth noting that immunotherapy has changed the landscape of cancer treatment. Immunotherapy is based on the knowledge of tumor escape mechanisms and aims to manipulate the immune system to reactivate the antitumor immune response and surmount the signaling pathways leading to escape. Early approaches to immunotherapy were focused on targeting cytokines that affected immune cell function. Nowadays, therapeutic alternatives are investigated to maneuver many facets of the immune system, such as immune checkpoint inhibitors (ICIs), adoptive cell therapy (ACT), oncolytic viruses, and cancer vaccines. Although, there are various approaches to tumor treatment, one of the major reasons for the unsatisfactory efficacy is that cancer cells, including those that are about to undergo death or are dying, can sculpt their surrounding microenvironment *via* secreting cytokines, chemokines, and other factors. An increasing number of researchers are beginning to investigate the diversity of cell death outputs and how they coordinate with the immune system. The immunogenic potential of dying tumor cells relies on their antigenicity and capacity to generate adjuvant signals commonly known as damage-associated molecular patterns (DAMPs) (1), and dying tumor cells that activate adaptive immune responses in immunocompetent hosts is also referred to as immunogenic cell death (ICD) (2). DAMPs emitted in the course of ICD, such as calreticulin (CALR), heat-shock proteins (HSPs), high mobility group box 1 (HMGB1), annexin A1 (ANXA1), secreted ATP, and type I interferons (IFNs), can be recognized by both the innate and adaptive immune systems *via* distinct pattern recognition receptors (PRRs) actuating chemoattraction, homing, activation, maturation, ultimately leading to the cross-presentation of tumor antigens to CD8^+^ cytotoxic T lymphocytes (CTLs) in the context of robust immune-stimulation (1). A tremendous amount of evidence suggests that when immune cells are present in the tumor microenvironment (TME), they promote tumor progression. Crosstalk between cancer cells and the proximal immune cells ultimately establishes soils that fosters tumor growth and recurrence. In this review, we will enumerate the current cell death occurred during cancer treatment, laying special emphasis on the link between different cell death modalities and tumor immune microenvironment (TIME), in order to figure out the underlying mechanisms that affect the results and regulation of immune system during cancer treatment.

## 2 Cell death modalities in cancer treatment

**Figure 1:**
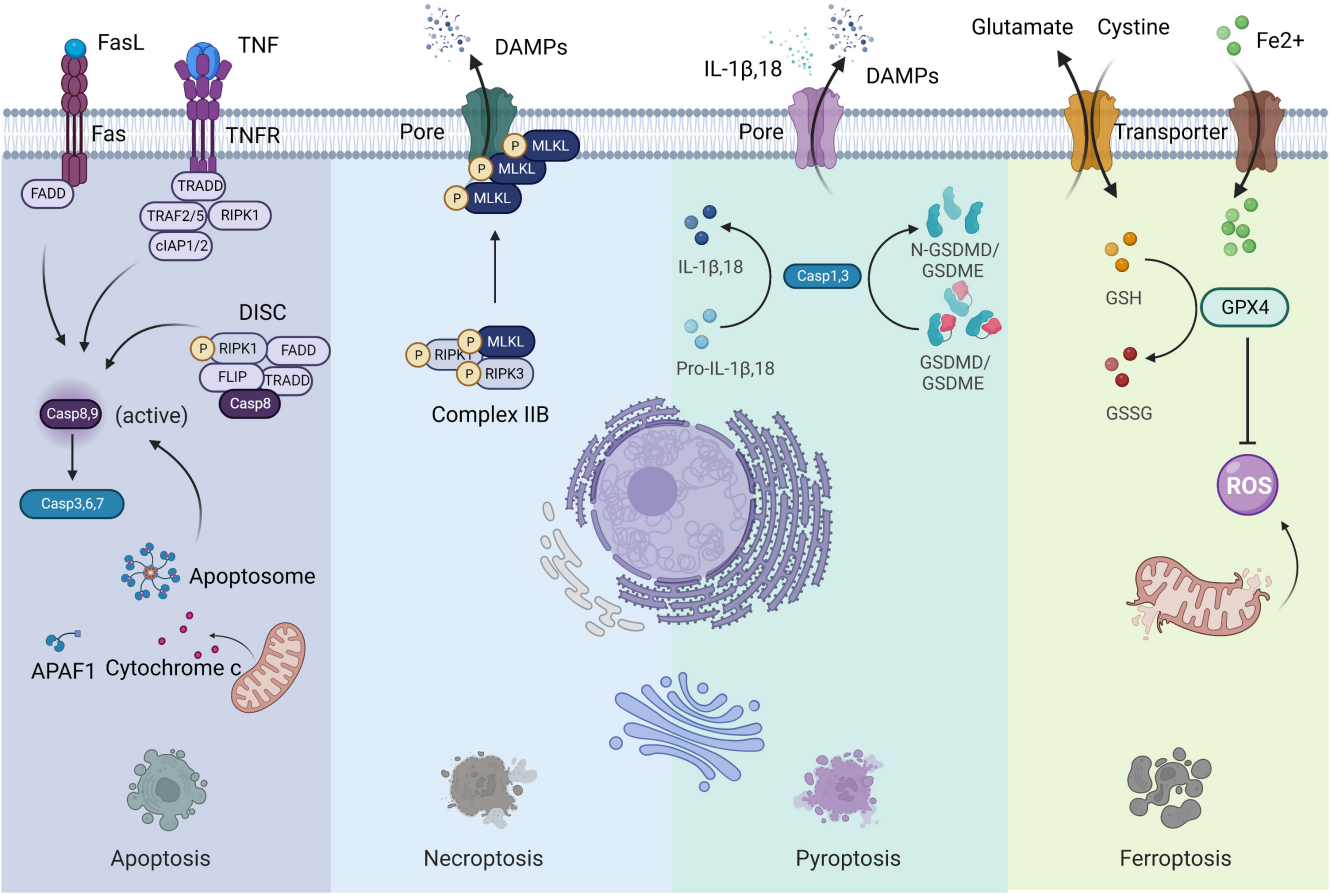
Four forms of regulated cell death. Left: Apoptosis; Middle to the left: Necroptosis; Middle to the right: Pyroptosis; Right: Ferroptosis.

Previous forms of cell death were classified into three main types based on macroscopic morphological changes: apoptosis (type I cell death), autophagy (type II cell death) and necrosis (type III cell death) (3). Since 2005, the cell death category system has been revamped by the Nomenclature Committee on Cell Death (NCCD), an organization formulating guidelines for the definition and interpretation of cell death from morphological, biochemical, and functional perspectives (2, 4). Fundamentally, cell death can be classified into accidental cell death (ACD) and regulated cell death (RCD) (2). ACD is uncontrolled and is a process caused by a biologically uncontrolled and harmful stimulus. Thus, ACD cannot be blocked by any medicinal or genetic interferences. However, cells undergoing ACD can release products toxic to surrounding cells, which may extend primary injury. In this context, the releasing molecules from cells undergoing ACD can be inhibited by particular Interventions, which may have a beneficial effect on prolonged disease outcomes. RCD is governed by a genetically encoded molecular machinery (2). Therefore, targeting RCD has promising potential in treating cancer. Although the forms of RCD are constantly evolving, only a few have been confirmed in the context of cancer. Hereby, we concentrate on the most pertinent and salient RCD patterns, namely apoptosis, necroptosis, pyroptosis, and ferroptosis (**Figure 1**). These four lytic forms of RCD have been discovered to trigger immune responses under the right circumstances, which are also known as ICD. Since our perception of cell death has radically changed, it is impractical to associate ICD to a specific type of RCD (2). Other cell death modalities have been extensively reviewed elsewhere (2).

### 2.1 Apoptosis

Apoptosis is the most extensively studied form of RCD characterized by morphological alterations of cell rounding, cytoplasmic shrinkage, plasma membrane blebbing and chromatin condensation, that participates in various physiological and pathophysiological processes (2). Apoptosis is usually divided into two forms: the intrinsic pathway (mitochondria) and the extrinsic pathway (death receptors). The intrinsic pathway is initiated by various internal traumas that eventuate in mitochondrial outer membrane permeabilization (MOMP), cytochrome c being released from the intermembrane space, and caspase 9 being activated by the formation of the apoptosome complex (2). The extrinsic pathway is mediated by death receptors, mainly Fas cell surface death receptor and TNF receptor superfamily members. When binding with ligands, the intracellular structural domains of these death receptors interact with adaptor proteins through the interplay of death structural and death effector domains, resulting in the activation of caspase 8 in a cytosolic complex called the death-inducing signaling complex (DISC). Both caspase 8 and caspase 9 are initiator caspases that can cleave and activate executioner caspases such as caspase 3, 6 and 7, leading to apoptosis. Surface-exposed calreticulin (ecto-CRT) and secreted ATP are crucial DAMPs for immunogenic apoptosis. It was reported that dying tumor cells induced by photodynamic therapy (PDT) underwent immunogenic apoptosis characterized by phenotypic maturation and functional stimulation of dendritic cells (DCs) as well as induction of a protective anticancer immune response (5). Early after PDT, cancer cells exhibited ecto-CRT and secreted ATP before displaying a biochemical feature of apoptosis, via a novel PERK-dependent subroutine that necessitate a competent secretory pathway and PI3K-mediated plasma membrane/extracellular trafficking (5).

### 2.2 Necroptosis

Necroptosis is considered as a regulated necrotic cell death modality driven by receptor-interacting kinase protein 1 (RIPK1) *via* forming complex IIB (necrotic body), requiring RIPK3 activity and being executed by mixed lineage kinase domain-like pseudokinase (MLKL) protein (6). Cancer cells undergoing necroptosis are characterized by cell membrane perforation and increased intracellular osmotic pressure, morphologically being cell rounding and swelling, and explosive rupture of the plasma membrane, resulting in releasing cell contents into extracellular space and exacerbating inflammatory response (7). Natural compounds, kinase inhibitors, irradiation and chemotherapy can induce necroptosis (8). Shikonin, an effective anticancer constituent, has been reported to inhibit both primary and metastatic osteosarcoma by inducing necroptosis (9). Necroptosis has been reported to promote or suppress cancer progression, contextualized in different types of cancers (8). Necroptotic pancreatic cancer cells can facilitate their migration and invasive abilities *via* CXCL5-CXCR2 axis (10). Necroptosis acts as an inflammatory mode of cell death with several signaling pathways activation, such as NF-κB and MAPK pathways, favoring cancer development (11).

### 2.3 Pyroptosis

Pyroptosis is an inflammatory caspase-dependent cell death modality that requires members of the gasdermin protein family, characterized by pore-formation, cell swelling and lysis, and release of proinflammatory mediators, including IL-1β, IL-18, ATP, and HMGB1 (12). Chemotherapy drugs can trigger tumor cell pyroptosis through caspase 3 cleavage of gasdermin E (GSDME) (13). Metformin treatment induces gasdermin D (GSDMD)-mediated pyroptosis of esophageal squamous cell carcinoma (ESCC) by impacting miR-497/proline-, glutamic acid- and leucine-rich protein-1 (PELP1) axis (14). The involvement of pyroptosis in cancer development appears to be contingent on the types of cancer, genetics, and the duration of pyroptosis induction (15). As the activation of pyroptosis is often accompanied by an inflammatory microenvironment, it is evident that pyroptosis contribute to cancer progression under some circumstances (15).

### 2.4 Ferroptosis

Ferroptosis is a newly defined form of RCD characterized by iron overload, lipid reactive oxygen species (ROS) accumulation, and lipid peroxidation (16). When cells undergo ferroptosis, mitochondria are smaller than normal, with a reduction or loss of mitochondrial cristae and rupture of the outer mitochondrial membrane, but the nuclear size is still normal, without chromatin condensation (17). Ferroptosis can be regulated *via* extrinsic and intrinsic pathways. The extrinsic pathway (transporter-dependent pathway) is triggered by repression of cell membrane transporters such as the cystine/glutamate antiporter system or by initiation of the iron transporters serotransferrin and lactotransferrin. The intrinsic pathway (enzyme-regulated pathway) is activated through blockade of intracellular antioxidant enzymes such as glutathione peroxidase 4 (GPX4) (18). However, the effector molecules of ferroptosis remain to be determined (19). The role of ferroptosis in tumorigenesis depends on the release of DAMPs and the activation of immune response within the TME. Many antitumor therapies have been reported to induce ferroptosis (17–19). Cisplatin induces ferroptosis through depleting intracellular glutathione (GSH) and inhibiting GPXs (20). The dysregulation of ferroptosis contributes to antitumor therapy resistance and failure.

Notably in 2022, Tsvetkov et al. discovered that excess intracellular copper could cause a proteotoxic stress response and ultimately cell death through direct binding to lipid acylated components of the tricarboxylic acid (TCA) cycle, leading to abnormal aggregation of lipid acylated proteins and loss of iron-sulfur (Fe-S) cluster proteins in the respiratory chain complex. The team found that this cell death is not blocked by apoptosis, necroptosis, pyroptosis and ferroptosis-related inhibitors, and therefore, is considered as a novel cell death mode and named as cuproptosis (21). This study shows that ferredoxin 1 (FDX1, a reductase that reduces Cu^2+^ to the more toxic Cu^1+^) and protein lipoylation are pivotal modulators of cuproptosis (21). However, there are few studies related to cuproptosis, and its overall regulatory mechanism in cancer remains to be further investigated.

## 3 The crosstalk between dying tumor cells and the tumor immune microenvironment

**Figure 2:**
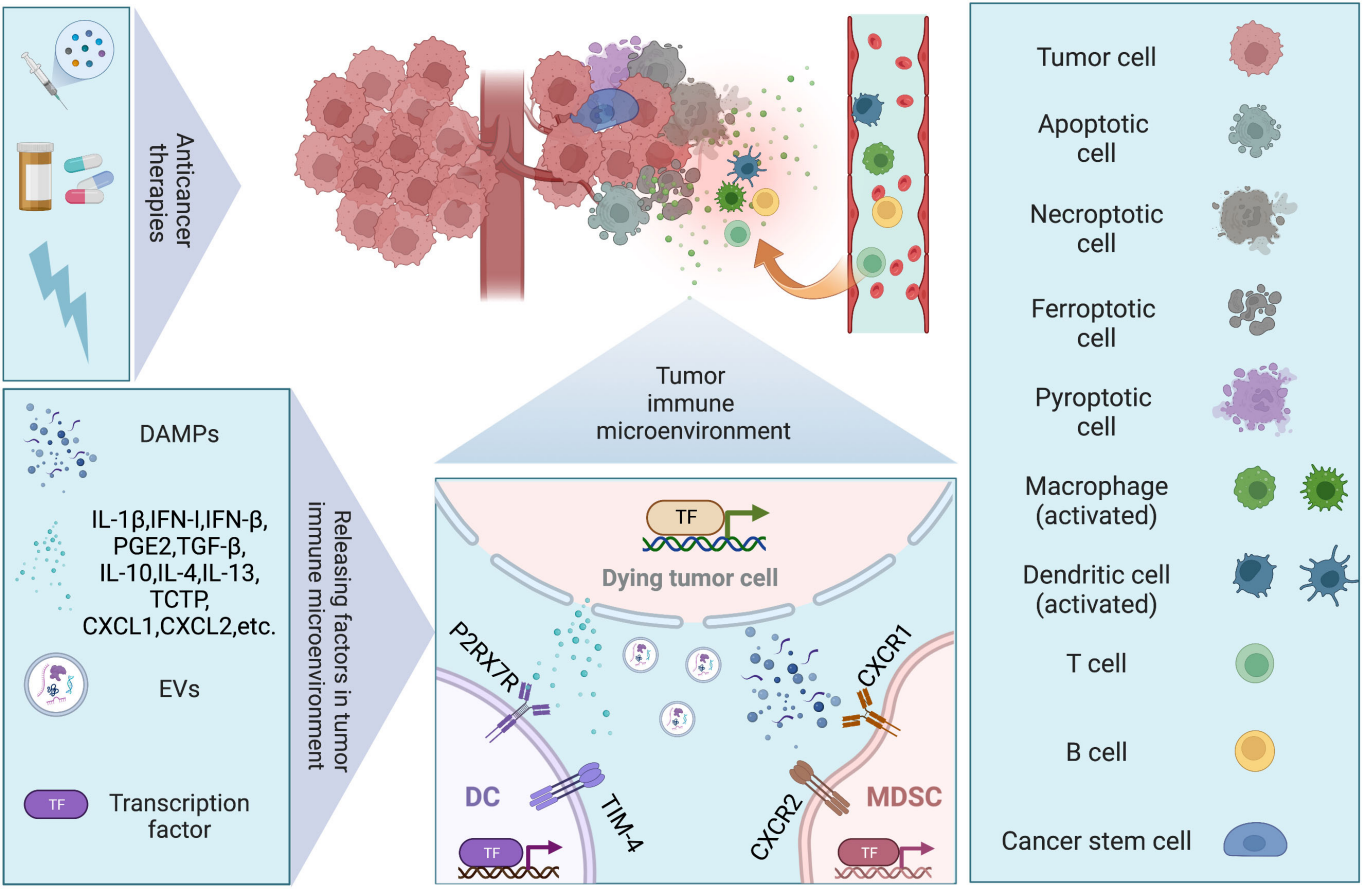
Dying tumor cells and tumor immune microenvironment. Conventional therapies (chemotherapy, targeted therapy, and radiotherapy) remain the mainstay of management in solid tumor, inducing different modalities of cell death. Regulated cell death (RCD), such as apoptosis, necroptosis, pyroptosis, and ferroptosis, have been discovered to trigger immune responses under the right circumstances, which are also known as immunogenic cell death (ICD). Under stresses, dying tumor cells release several factors including damage-associated molecular patterns (DAMPs), chemokines, cytokines, and extracellular vesicles (EVs), to modulate immune system *via* facilitating cancer stem cell repopulation, governing inflammation, promoting angiogenesis, reprogramming metabolites.

Antitumor therapy achieves its ultimate therapeutic goal by causing cell death, but many studies have now found that tumor cell death can also promote tumor recurrence or enhance the therapeutic resistance of tumor cells by affecting the TME. The TME consists of immune cells, stromal cells, blood vessels, lymphatic vessels, nerve terminals and extracellular matrix (ECM) immunomodulating the microenvironment through various signaling molecules that continuously remodel local immunity. The composition of the TME is a key factor in tumor-host interactions. The tumor immune microenvironment (TIME), on the other hand, emphasizes the different cell populations of the immune system and their interactions in the TME, and is of interest for its key role in carcinogenesis, cancer progression and response to therapy (22). In this section, we will present how cell death affects the TIME, both directly and indirectly (**Figure 2**).

### 3.1 Immune cells

Phagocytosis of dying tumor cells by DCs results in cross-presentation of cell-associated antigen, and the priming of CD8^+^ T cells, which has been implicated in the maintenance of immune homeostasis. When mice were immunized with cells that experienced RIPK3-mediated necroptosis, CD8^+^ T cells cross-priming was significantly higher compared to immunization with caspase-8-mediated apoptotic cells (23). Both RIPK1 signaling and NF-κB activation within the dying tumor cells are required for efficient cross-priming and antitumor immunity (23). It was demonstrated that ATP released by dying cancer cells activated P2RX_7_ receptors on DCs and induced the nucleotide-binding oligomerization domain (NOD)-like receptor family, pyrin domain containing-3 protein (NLRP3) inflammasome, linking the innate and adaptive immune responses against dying cancer cells *via* secretion of IL-1β (24). Cytosolic DNA released by dying tumor cells induces the production of type I IFN (IFN-I) by DCs through cGAS-STING pathway, contributing to antitumor immunity (25). However, it is also reported that DAMPs released from dying tumor cells upregulate T cell immunoglobulin and mucin domain protein-4 (TIM-4) expression levels on TAMs and DCs, which activating autophagy and impeding anti-tumor immunity *via* TIM-4-AMPKα1 interaction (26). Conventional type 1 dendritic cells (cDC1) are vital for antitumor immunity and the accumulation of cDC1 relies on chemoattractants CCL5 and XCL1 produced by NK cells (27). Dying tumor cells produced prostaglandin E2 (PGE_2_) to evade from the NK cell-cDC1 axis by reducing NK cell viability and chemokine production, as well as by downregulating chemokine receptor expression in cDC1 (27). MDSCs are a heterogenous group of myeloid cells that are highly suppressive to antitumor lymphocyte function and trafficking to tumor (28). Two major subsets of MDSCs have been identified in humans: polymorphonuclear MDSCs (PMN-MDSCs) and monocytic MDSCs (M-MDSCs) (28, 29). Translationally controlled tumor protein (TCTP) released by dying tumor cells induces expression of CXCL1 and CXCL2, which then recruits PMN-MDSC population to TIME and exerts immunosuppressive function (29). The resultant accumulation of PMN-MDSCs also strongly attenuates the activation and aggregation of CD8^+^ T and NK cells in the TIME (29–31). Inhibition of TCTP averts PMN-MDSC accumulation and tumor growth (29). Additionally, the immunomodulatory activity of irradiation-induced cell death varies. High non-fractionated radiation doses above the threshold (ranging from 12 to 18 Gy in different cancer cells) induce the expression of the DNA exonuclease Trex1, which degrades cytoplasmic DNA and mitigates the production of IFN-I and the immunostimulatory effects on DCs (32, 33). In contrast, radiation given in fractionated doses below the dose threshold for Trex1 induction stimulates dying tumor cells to release IFN-β cytokine, which recruits Batf3-dependent DCs and activates anti-tumor CD8^+^ T cells (33).

### 3.2 Cancer stem cells

Emerging evidence demonstrates that cancer development and progression are associated with a tumor subpopulation, termed cancer stem cells (CSCs). CSCs not only show treatment resistance, but also regulate the immune system in order to avoid being eliminated by immune cells (34). CSCs have been proved to facilitate macrophage differentiation toward an immunosuppressive M2 phenotype by secreting macrophage-colony stimulating factor (M-CSF), which depends on the constitutive activation of interferon-regulatory factor 5 (IRF5) (35). CSCs also promote the production of IL-6 and IL-1β to mediate Th17 cell differentiation, as well as IL-8 and TNF-α to stimulate neutrophil tumor infiltration and angiogenesis (35–37). In turn, TAMs promote the expansion of CSCs *via* IL-6 and STAT3 signaling, which stimulate further cytokine production, generating a positive feedback loop for CSC self-renewal (38, 39). Moreover, CSCs secrete several cytokines to exert inhibitory effects on immune cells in TME, including TGF-β, IL-10, IL-4 and IL-13 (40). Although chemotherapy effectively elicited apoptosis, associated PGE_2_ released by these dying tumor cells could paradoxically facilitate neighboring CSC repopulation (41). Blockade of PGE_2_ production using cyclooxygenase-2 (COX2) inhibitors abrogated this effect (41).

### 3.3 Inflammation

Several studies have highlighted the interconnections between cell death and inflammatory signal transduction (42). Inflammasomes are cytoplasmic multimeric protein complex that provide molecular platforms for activation of caspase-1, contributing to pyroptosis (43). The formation of inflammasome requires cytosolic sensing of pathogen-associated molecular patterns (PAMPs) or DAMPs by the nucleotide-binding domain and leucine-rich repeat receptors (NLRs) or absent in melanoma 2 (AIM2)-like receptors (ALRs) (43). Various innate immune receptors also assemble inflammasome complexes (42). It is widely believed that inflammasomes either favor or suppress carcinogenesis in a highly context-dependent manner. Among NLRs, NLRP3 senses the broadest array of stimuli and participates in adaptive immunity. It is testified that NLRP3 inflammasome drives a T-cell response towards dying tumor cells (24). However, the detrimental effect of NLRP3 is observed when mice are treated with DC vaccination against the poorly immunogenic melanoma cell line B16-F10, *via* enhancing the accumulation of MDSCs (44). NLRP3 is also an important suppressor of NK cell-mediated responses to carcinogen-induced tumors and metastases (45).

### 3.4 Angiogenesis

A functional vascular network delivers continued oxygen and nutrients to cells, however, the vasculature of tumor is structurally and functionally abnormal, such as poorly organized vasculature, tortuous and dilated vessels with uneven diameter, excessive branches and shunts (46). The impaired vessel perfusion and high vascular permeability increase tissue hypoxia, acidosis, and necrosis, which could reprogram the resident macrophages into a pro-tumorigenic and immunosuppressive phenotype (47, 48). Also, the hypoxic and acidic microenvironment could up-regulate programmed cell death ligand 1(PD-L1) on MDSCs, DCs and cancer cells, polarize TAMs to the immune inhibitory M2-like phenotype to suppress T-effector cell function. Chronic hypoxia and microenvironment acidity contribute to immune evasion by inducing T cells differentiation into immunosuppressive Tregs (49, 50). Actually, hypoxia is often found in solid tumors due to the rapid growth of cancer cells (51). Hypoxia could influence monocytic cells with respect to morphology, antigen expression, and cytokine secretion, and to induce various genes including vascular endothelial growth factor (VEGF), fibroblast growth factor-3 (FGF-3), BCL2-interacting killer (BIK), and MMPs (52–54). Hypoxia-inducible factors (HIFs) are major transcription factors for cancer cells to adapt hypoxic stress and the activation of HIF pathway results in angiogenesis (55). VEGF family members play important roles in HIF induced angiogenesis. There is mounting evidence to support the perspective that angiogenesis and inflammation are interdependent (56, 57). It is well established that cancer cells are able to secrete pro-angiogenic factors as well as inflammatory mediators (37). Our previous studies have shown that apoptotic tumor cells mediate proangiogenic response *via* caspase 3/NF-κB/COX2/PGE_2_ and caspase 3/p-eIF4E/VEGF-A pathways after irradiation (58). Additionally, normalizing blood vessels with anti-angiogenic therapy normalizes tumor vasculature, creates a more uniform distribution of perfused tumor vessels, and promotes infiltration of T effector cells while inhibiting the accumulation of MDSC (56, 57). Normalized vasculature also alleviates the hypoxia and acidity in TIME, which in term polarizing TAMs to an immunostimulatory M1-like phenotype (56, 57).

### 3.5 Metabolic reprogramming

Emerging evidence has pointed out that the immune response is correlated with dramatic modifications in tissue metabolism, such as nutrients exhaustion, oxygen depletion, and increased accumulation of reactive nitrogen and oxygen intermediates (59). Given their coexistence, cancer cells and immune cells are in the competition for the local resources available in the microenvironment. When cancer cells underwent dying process, the nutrients competition would still exist. The dying tumor cells either utilized the nutrients around themselves to survival or against demise, or sacrificed themselves to support remnant tumor cells proliferation or escape from immune clearance. It has been reported that apoptotic cells can release metabolites to activate specific programs in neighboring cells (60). Cancer cell death leads to a local ionic imbalance within the TIME as well. In necrotic areas, dying tumor cells release a high number of cations, specifically potassium ions, causing functional starvation of the tumor-infiltrating T cells in parallel to suppression of AKT/mTOR phosphorylation (61). Overabundance of potassium in the TIME also limited T cell effector function by promoting autophagy and metabolic reprogramming, while decreased histone acetylation at effector and exhaustion loci, which in turn generating T cell stemness with improved persistence, engraftment, self-renewal, multipotency, and tumor clearance (62). Lactic acid, a metabolic product of cancer cells, is reported to act as an epigenetic regulator and elicit M2 macrophage polarization through epigenetic reprogramming (63). It binds straight to the histone lysine lactylation (Kla) sites and the transcription of downstream genes is stimulated, which then invokes M2 polarization and reinforces inflammation-independent biological pathways (63). G protein-coupled receptor 81 (GPR81) is a lactic acid receptor found on both immune cells and cancer cells (64). Its activation in cancer cells promotes proliferation, therapy resistance, and enhanced expression of PD-L1 (65–67). Its activation on DCs is associated with decreased cAMP, IL-6, IL-12, and prevents tumor-specific antigen presentation (67). The depletion of glutamine, arginine, and tryptophan also show immunosuppressive effects. It is demonstrated that cancer cells could express indoleamine 2,3-dioxygenase (IDO), an enzyme that catalyzing tryptophan (TRP) and suppresses immune response within the TME (68). Gut microbiota impacts the amenability of some tumor types to therapies by influencing regulatory aspects of the immune response. More clearly, the microbiome has been reported to play a critical role in determining DC functionality (69, 70).

### 3.6 Extracellular vesicle

Extracellular vesicles (EVs) are a heterogeneous class of cell-derived membranous structures produced by all cells that contain proteins, lipids and genetic material (71). EVs are essential mediators of cell-cell communication and can be broadly divided into two distinct classes: exosomes and microvesicles (MVs) (71). Tumor-derived exosomes are involved in many stages of tumor progression, including angiogenesis (72), therapy resistance (73), invasion and metastasis (74), and immune evasion (75). Dying tumor cells treated by antitumor agent topotecan (TPT) could release exosomes that contain DNA to activate DCs *via* a STING-dependent pathway (76). Exosomes derived from tunicamycin (TM)-treated cancer cells contain abundant miR-23a-3p, which inhibit PTEN expression and subsequently elevated phosphorylated AKT and PD-L1 expression in macrophages, thus helping cancer cells to escape from antitumor immunity (77). MVs arise *via* direct outward budding and fission of the plasma membrane and participate in cell-to-cell communication, immune regulation, and tumor progression (71). Dying tumor cells could release MVs loaded with PD-L1 to suppress the function of T cells and induce M2 phenotype macrophages *via* TBK1/STAT6 and AKT/mTOR signals, contributing to the suppressive TIME (78).

## 4 Immunotherapies

While conventional therapies remain the mainstay of management in solid tumor, immunotherapy is rapidly being incorporated with these therapies to prolong patient survival (79). Immunotherapies are currently confined to those antibodies targeting PD-1/PD-L1 and cytotoxic T-lymphocyte-associated protein 4 (CTLA-4). However, these ICIs often show transient efficacy, especially in microsatellite stable tumors which often present minor genetic abnormalities and possess limited antigenic capacity (80). The immunosuppressive TME also contributes to the restricted effects of ICIs. It is reported that the response rate to single agent PD-1 blockade ranges from 40% to 70% in cancers such as melanoma or Hodgkin’s lymphoma, while limited to 10-25% in most other cancer types (81–83). Moreover, these patients who initially respond to ICIs can eventually have disease progression. Thus, strategies to improve efficacy are urgently needed. Combination strategies have been formulated to confront the challenge of immunotherapy resistance and broaden the responding population (**Table 1**). For instance, immunotherapy has been combined with chemotherapy in lung, breast and gastric cancer, with TKIs in RCC and bladder cancer (83). In the CheckMate 816 study (NCT02998528), neoadjuvant immunotherapy combined with chemotherapy was associated with significantly longer event-free survival than chemotherapy alone in patients with resectable NSCLC (31.6 months vs. 20.8 months) and a higher proportion of patients with pathologic complete responses (24% vs. 2.2%). The addition of immunotherapy to neoadjuvant chemotherapy did not increase the incidence of adverse events or impede the feasibility of surgery (84). Ceralasertib has excellent antitumor activity as an oral kinase inhibitor in combination with immunotherapy (NCT03780608), with a durable response in patients with advanced gastric cancer (85). During study treatment, investigators also found an upregulation of cytoplasmic DNA-induced innate immune responses, activation of intratumoral lymphocytes and expansion of circulating tumor-reactive CD8^+^ T cell clones in responders (85). Of course, some clinical studies have concluded that combination therapy does not improve the effectiveness of immunotherapy very well. A multicenter randomized phase II clinical study found that radiotherapy did not increase the response to PD-L1 plus CTLA-4 combination inhibition in NSCLC patients resistant to PD(L)-1 therapy (NCT02888743) (86). Thus, clinically, the effectiveness of immunotherapy needs to be further clarified, and the mechanism of limited effect of combined immunotherapy and non-response to immunotherapy may be related to TIME, and more basic research on molecular mechanism is expected to follow.

**Table 1:** Summary some of the clinical trials testing combinations of immunotherapy with therapeutic agents that may target apoptosis, necroptosis, pyroptosis, and ferroptosis.

Combination Strategies	Therapeutic agents	Cancer type	Effects on cancer cell death	Clinical trial phase	Clinicaltrials.gov ID
Chemotherapy	nivolumab plus platinum-based chemotherapy	NSCLC	Apoptosis/necroptosis/pyroptosis/ferroptosis	III	NCT02998528
	carboplatin plus paclitaxel with camrelizumab	Advanced squamous NSCLC	Apoptosis/necroptosis/pyroptosis/ferroptosis	III	NCT03668496
	Toripalimab+paclitaxel plus cisplatin	Advanced ESCC	Apoptosis/necroptosis/pyroptosis/ferroptosis	III	NCT03829969
	Tislelizumab plus concurrent chemoradiotherapy	Advanced ESCC	Apoptosis/necroptosis/pyroptosis/ferroptosis	III	NCT03957590
	Gemcitabine and cisplatin plus durvalumab +tremelimumab	Advanced BTC	Apoptosis/necroptosis/pyroptosis/ferroptosis	II	NCT03046862
	Durvalumab plus platinum-pemetrexed chemotherapy	Unresectable pleural mesothelioma	Apoptosis/necroptosis/pyroptosis/ferroptosis	II	NCT02899195
	FOLFOX6+pembrolizumab	Advanced CRC	Apoptosis/necroptosis/pyroptosis/ferroptosis	Ib	NCT02375672
	Carboplatin+etoposide +adebrelimab	ES-SCLC	Apoptosis/necroptosis/pyroptosis/ferroptosis	III	NCT03711305
Radiotherapy	Radiotherapy+durvalumab plus tremelimumab	Metastatic NSCLC	Apoptosis/necroptosis/pyroptosis/ferroptosis	II	NCT02888743
	Nivolumab and ipilimumab+SBRT	Stage IV NSCLC	Apoptosis/necroptosis/pyroptosis/ferroptosis	I	NCT03223155
	Radiation+ipilimumab and nivolumab	Metastatic MSS CRC	Apoptosis/necroptosis/pyroptosis/ferroptosis	II	NCT03104439
	Toripalimab with concurrent chemoradiotherapy	Locally advanced cervical cancer	Apoptosis/necroptosis/pyroptosis/ferroptosis	II	NCT05084677
Targeted therapy	Olaparib+durvalumab + tremelimumab	Solid cancers	Apoptosis/ferroptosis	II	NCT04169841
	Ceralasertib plus durvalumab	Metastatic melanoma,AGC	Apoptosis	II	NCT03780608
	Cobimetinib+atezolizumab	BTC	Apoptosis	II	NCT03201458
	Cabozantinib+atezolizumab	mCRPC	Apoptosis	Ib	NCT03170960
	Apatinib+camrelizumab+eribulin	Advanced TNBC	Apoptosis/pyroptosis/ferroptosis	II	NCT04303741
	Famitinib+camrelizumab	Platinum-resistant ROC	Apoptosis	II	NCT03827837
	Regorafenib+avelumab	advanced BTCs	Apoptosis	II	NCT03475953

NSCLC, non-small cell lung cancer; ESCC, esophageal squamous cell carcinoma; BTC, biliary tract cancer; CRC, colorectal cancer; ES-SCLC, extensive-stage small-cell lung cancer; MSS, microsatellite stable; AGC, advanced gastric cancer; mCRPC, metastatic castration-resistant prostate cancer; TNBC, triple-negative breast cancer; ROC, recurrent ovarian cancer.

## 5 Conclusions and perspectives

Effective elimination of cancer cells has always been the mainstay and goal of clinical cancer treatment. However, no matter what treatment options are adopted, there are still residual cancer cells surviving and even proliferating to form new tumor masses. In this process, TIME has been proven to play an important role. And there are multiple molecular interactions between cancer cell death and TIME, which affect the efficacy of immunotherapy to an extent. Thus, investigation and targeting of molecules at the crossroad of dying tumor cells and host immune system may provide new therapeutic opportunities in the area of cancer treatment especially immunotherapy.

## Author contributions

SJH and JC conceived the manuscript. SJH wrote the manuscript and designed the figures and tables. JC and QH reviewed and revised the manuscript. All the authors contributed to the manuscript have read and agreed to the published version.
